# Affinity Enhancement by Ligand Clustering Effect Inspired by Peptide Dendrimers−Shank PDZ Proteins Interactions

**DOI:** 10.1371/journal.pone.0149580

**Published:** 2016-02-26

**Authors:** Jiahui Liu, Miao Liu, Bo Zheng, Zhongping Yao, Jiang Xia

**Affiliations:** 1 Department of Chemistry, Centre of Novel Biomaterials, The Chinese University of Hong Kong, Shatin, Hong Kong, China; 2 Department of Applied Biology & Chemical Technology, The Hong Kong Polytechnic University, Kowloon, Hong Kong, China; University of Quebec at Trois-Rivieres, CANADA

## Abstract

High-affinity binders are desirable tools to probe the function that specific protein−protein interactions play in cell. In the process of seeking a general strategy to design high-affinity binders, we found a clue from the βPIX (p21-activated kinase interacting exchange factor)−Shank PDZ interaction in synaptic assembly: three PDZ-binding sites are clustered by a parallel coiled-coil trimer but bind to Shank PDZ protein with 1:1 stoichiometry (1 trimer/1 PDZ). Inspired by this architecture, we proposed that peptide dendrimer, mimicking the ligand clustering in βPIX, will also show enhanced binding affinity, yet with 1:1 stoichiometry. This postulation has been proven here, as we synthesized a set of monomeric, dimeric and trimeric peptides and measured their binding affinity and stoichiometry with Shank PDZ domains by isothermal titration calorimetry, native mass spectrometry and surface plasmon resonance. This affinity enhancement, best explained by proximity effect, will be useful to guide the design of high-affinity blockers for protein−protein interactions.

## Introduction

Clustering of multiple copies of ligands is a common strategy to attain high-affinity interactions in biology [1–4]. Ligands can be clustered in linear form, such as polyproline peptide 33-mer that binds with enhanced affinity with the protein receptor DQ2, a hallmark of gluten-induced antigen presentation [5, 6]. Ligands can also be clustered in a dendrimeric form. One intriguing example of dendritically clustered ligand is the protein βPIX (p21-activated kinase interacting exchange factor), which binds to Shank PDZ domain [7–9].

**Fig 1:**
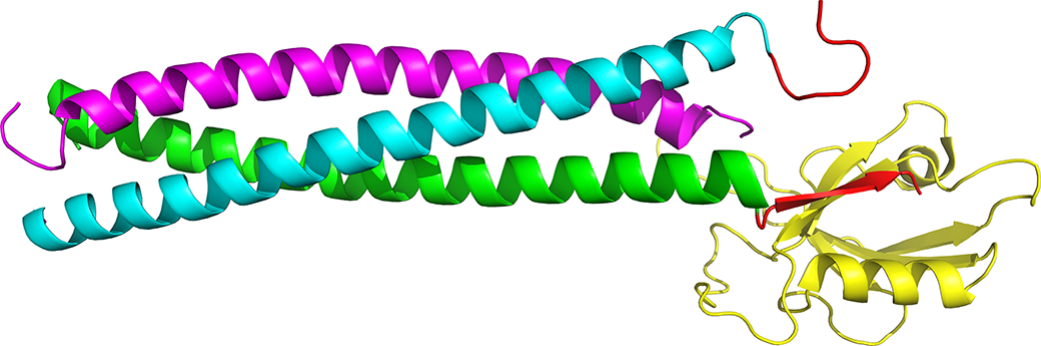
The structure ofβPIX trimer bound with Shank PDZ (PDB ID 3L4F). [9] Peptide binding motif ofβPIX is indicated by the red arrow.

Shank protein, a scaffolding protein in PDZ that mediates the organization of synaptic protein complexes, contains one PDZ domain, Shank PDZ [10–14]. Like all other canonical PDZ domains, Shank PDZ (having subtypes such as Shank1 PDZ, Shank2 PDZ and Shank3 PDZ) is composed of ~90 amino acids, folds into a structure of a six-stranded β-sandwich and two α-helices, and recognizes the extreme C terminal sequences of other proteins [15]. Among the various ligands, βPIX, a guanine nucleotide exchange factor used by Rho GTPase family members Rac1 and Cdc42, binds to Shank PDZ through a PDZ-binding sequence at its extreme C terminus; this interaction promotes synaptic accumulation of βPIX for Rac1 and Cdc42 [7–9]. Interestingly, a leucine-zipper sequence was found N terminal to the PDZ-binding site, which trimerizes as a parallel coiled coil and assembles a triply clustered peptide ligand with three PDZ-binding sites closely located in a limited space. Crystallographic work as well as binding analysis found the βPIX trimer holds 1:1 binding stoichiometry with Shank1 PDZ (1 trimer with 1 PDZ) (Fig 1) [9].

Intrigued by the triply clustered ligand geometry yet with 1:1 binding stoichiometry, we set out to design peptide dendrimers for Shank PDZ as mimics for the clustered ligand in βPIX, to explore the affinity and the stoichiometry of the binding interaction, and to examine whether this ligand clustering effect can guide the design of high-affinity binders for protein-protein interactions.

## Materials and Methods

### Peptide Synthesis and Purification

Attachment of the first leucine on Wang-ChemMatrix resin was carried out by 1-(Mesitylene-2-sulfonyl)-3-nitro-1,2,4-triazole (MSNT) method: Fmoc-Leucine (2 eq.) and N-methylimidazole (4 eq.) were dissolved in DCM, followed by MSNT (2 eq.), the swelled resin were then stirred with mixture solution for 4 hours, the uncoupled hydroxyl groups were capped by acetic anhydride (2 eq.) for 30 minutes. Then the procedure follows solid phase peptide synthesis technique based on the Fmoc chemistry.

Then resin were deprotected by piperidine/DMF (20% v/v) for 30 minutes, and then stirred with amino acid (5 eq.) activated by HBTU (5 eq.), HOBt (5 eq.) and DIEA (10 eq.) in DMF for 45 minutes. After the peptide sequence was completed, N terminal amino groups were acetylation by acetic anhydride (2 eq.) for 30 minutes. Peptides were cleaved from the resin by a cleavage cocktail (trifluoroacetic acid /1,2-ethanedithiol/water/triisopropylsilane/phenol 82.5: 2.5:5:5:5, v/v) for 4 h at room temperature.

The crude peptides were precipitated in ice-cold diethyl ether, pelleted by centrifugation, dissolved in 1:1 v/v acetonitrile/water. The peptides were purified by reserve-phase HPLC, and then lyophilized and stored at -20°C. The identity and purity of the peptides were confirmed by MS spectra and HPLC.

Dendrimeric ligands were synthesized from pure monomeric cysteine-containing peptides. For dimeric peptide ligand **p2**, the monomeric motif CGSSGDPAWDETNL was dissolved in phosphate buffer (50 mM sodium phosphate, 5 mM EDTA, pH 6.5), and bis-functional linker 1,8-bis(maleimido)diethylene glycol (BM[PEG]_2_) dissolved in DMF was added into peptide solution as 1:2.5 ratio, and the solution was stirred 1 h at room temperature. Changing the linker BM[PEG]_2_ to 1,11-bis(maleimido)triethylene glycol (BM[PEG]_3_), dimeric peptide ligand with different linker length was synthesized. For trimeric peptide ligand **p3**, tri-functional linker tris-(2-maleimidoethyl) amine (TMEA) was added as 1:4 ratio. The reactions were monitored by HPLC and the products were purified by a semi-preparative column and confirmed by MS spectra. The concentration of peptide solution was quantified by UV-Vis absorption spectrum using ε_280nm_ = 5690 cm^-1^ M^-1^ for monomer ligand, ε_280nm_ = 11380 cm^-1^ M^-1^ for dimeric ligand, ε_280nm_ = 17070 cm^-1^ M^-1^ for trimeric ligand.

### Protein expression and purification

Gene encoding the Shank1 PDZ (Swiss-Prot accession number *Q9WV48*.*1*, residues 653–765) was synthesized by GenScript Inc., and digested by Nde I and Xho I restriction enzymes and ligated into pET28a vector to generate pET28-Shank1 PDZ plasmid (S1A Fig). The plasmid pET.M.3C Shank3 PDZ (Swiss-Prot accession number *Q4ACU6*.*3*, residues 533–665) was a gift from Prof. Mingjie Zhang’s group from Division of Life Science, Hong Kong University of Science and Technology. The recombinant plasmid pET28-Shank1 PDZ and pET.M.3C Shank3 PDZ were transformed into *E*. *coli* BL21 cells for protein production. Mono-clones were pre-cultured overnight at 37°C, and then cultures were added to new media, shaken at 37 °C until optical density at 600 nm reached 0.6–0.8. Then isopropyl β-D-1-thiogalactopyranoside was added at final concentration 1 mM to induce protein overexpression, the culture was shaken overnight at 16°C.

Cell culture was harvested and re-suspended in binding buffer (50 mM sodium phosphate, 300 mM NaCl, 10 mM imidazole, 4 mM β-mercaptoethonol, pH 8.0), and then lysed by a sonicator. The cell lysate were centrifuged, and the supernatant was bound to binding buffer equilibrated HisTrap HP column, and then eluted by elution buffer (50 mM sodium phosphate, 300 mM NaCl, 500 mM imidazole, 4mM β-mercaptoethonol, pH 8.0). The fractions containing the purified his-tagged protein were collected, exchanged buffer and concentrated, and store in the storage buffer (50 mM sodium phosphate, 200 mM NaCl, pH 7.4) at 4°C. The concentration of protein was quantified by UV-Vis absorption spectrum using ε_280nm_ = 8480 cm^-1^ M^-1^ for Shank1 PDZ and ε_280nm_ = 11460 cm^-1^ M^-1^ for Shank3 PDZ.

### Isothermal titration calorimetry

Prior to use, lyophilized peptides were dissolved in the storage buffer (50 mM sodium phosphate, 200 mM NaCl, pH 7.4). All the peptides and protein solution were filtered through 0.1 μm membrane, and their concentrations were adjusted according to experiments requirement: except for the binding pair **p1** ligand and Shank1 PDZ, the proteins concentration were between 30–40 μM, ligands concentration were between 300–400 μM; and for the binding pair **p1** ligand and Shank1 PDZ, the affinity was very low so that protein concentration was adjusted to about 315 μM and **p1** ligand concentration was adjusted to about 3.14 mM to get accuracy affinity data. All the solutions were extensively degassed just before titration. Titrations were performed at 25°C by MicroCal VP-ITC (Malvern), and data were analyzed by Origin software (Origin Lab) and fitted to single-binding-site model.

### Surface Plasmon Resonance

Prior to use, lyophilized peptides were dissolved in the storage buffer (50 mM sodium phosphate, 200 mM NaCl, pH 7.4) at high concentration and diluted to required concentration using running buffer (HBS-EP: 0.01 M HEPES pH 7.4, 0.15 M NaCl, 3 mM EDTA, 0.005% v/v Surfactant P20). All the peptides and protein solution were filtered through 0.1 μm membrane, and their concentrations were adjusted according to experiments requirement: for ligand **p1**, concentration series of 10.8 μM, 21.6 μM, 43.2 μM, 86.5μM, 173μM, 346μM were used; for ligand **p2**, concentrations series of 10 μM, 20 μM, 40 μM, 80 μM, 160 μM, 320 μM were used; for ligand **p3**, concentrations series of 2.63 μM, 5.27 μM, 10.5 μM, 21.1 μM, 42.1 μM, 84.3 μM were used. All sample solution and running buffer were extensively degassed just before titration. Shank1 PDZ protein was coupled on CM5 chip using EDC/NHS method.

Running were performed at 25°C on Biacore 3000 (GE), and data were processed by BIAevaluation software and fitted to steady state affinity model and 1:1 Langmuir binding model. 1:1 Langmuir binding model which show kinetics data was analyzed by BIAevaluation software. Steady state affinity model which show affinity data was analyzed by Origin software using the following equation:

$$R_{eq}\;=\;\frac{K_{A}CR_{max}}{1+\;K_{A}Cn}$$

### Native mass spectra

Prior to the experiment, the protein solution was extensively dialyzed at 4°C against 150mM ammonium acetate. During the dialysis, Shank1 PDZ yielded no precipitate, while Shank3 PDZ was largely precipitated out. Increasing the concentration of ammonium acetate to 200–500 mM couldn’t alleviate precipitation. Lyophilized peptides were also dissolved in the 150 mM ammonium acetate. All the peptides and protein solution were filtered through 0.1 μm membrane, and their concentrations were adjusted according to experiments requirement. The protein and peptide were mixed as the corresponding ratio and incubated at room temperature for 20 minutes.

Positive ion electrospray ionization mass spectra were acquired on a Micromass Q-Tof micro mass spectrometer (Waters), with a nanoESI source using EconoTips. The capillary voltage was adjusted in the range of 1.2–2.2 kV until optimal ion signals were obtained. The cone voltage of the Q-TOF was set at 20 V, and the ion source temperature was set at 40°C.

## Results and Discussion

### Enhanced binding affinity from monomer to trimer

**Fig 2:**
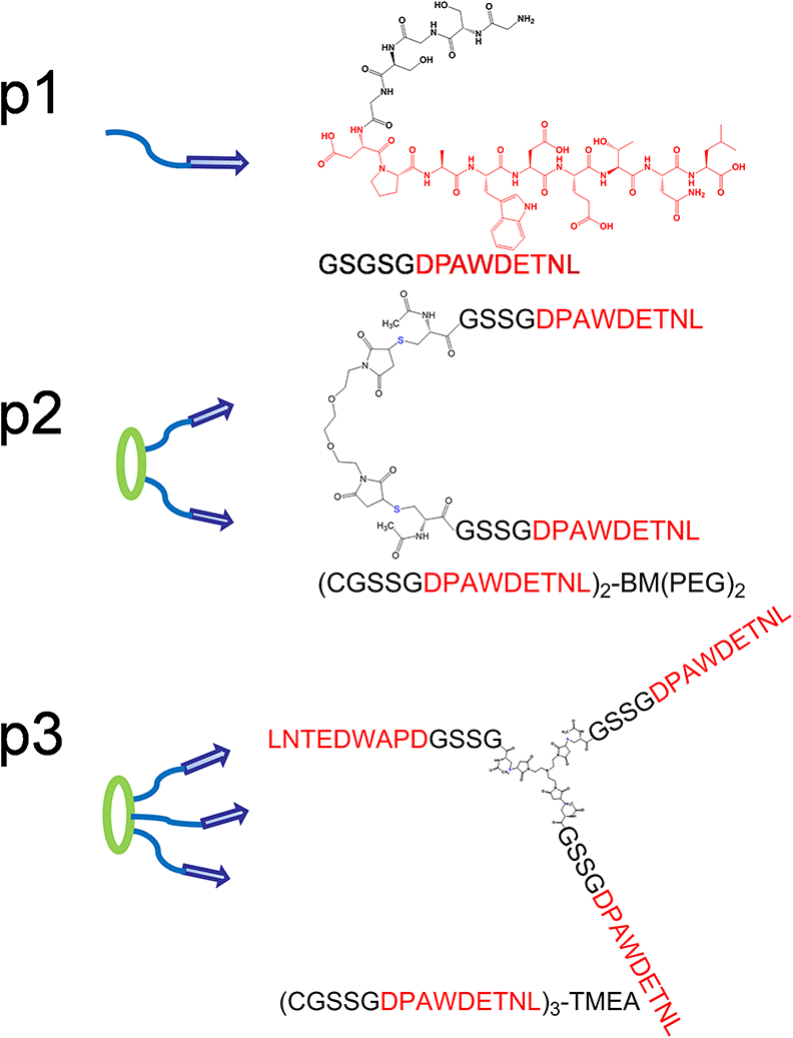
Peptide dendrimers for Shank PDZ as mimics for the clustered ligand in βPIX. The design of monomeric, dimeric and trimeric peptides **p1**, **p2** and **p3** to mimic the trimer structure ofβPIX.

A covalently linked peptide trimer is a superior mimic of ligand cluster in βPIX trimer [16, 17], because the triple helical coiled coil can dissociate at low concentration, which complicates the analysis of peptide−protein binding complexes. We synthesized trimeric and dimeric peptide by linking the monomer through divalent and trivalent linkers BM(PEG)_2_ and TMEA through maleimide-thiol Michael addition reaction (Figs 2 and S2).

**Fig 3:**
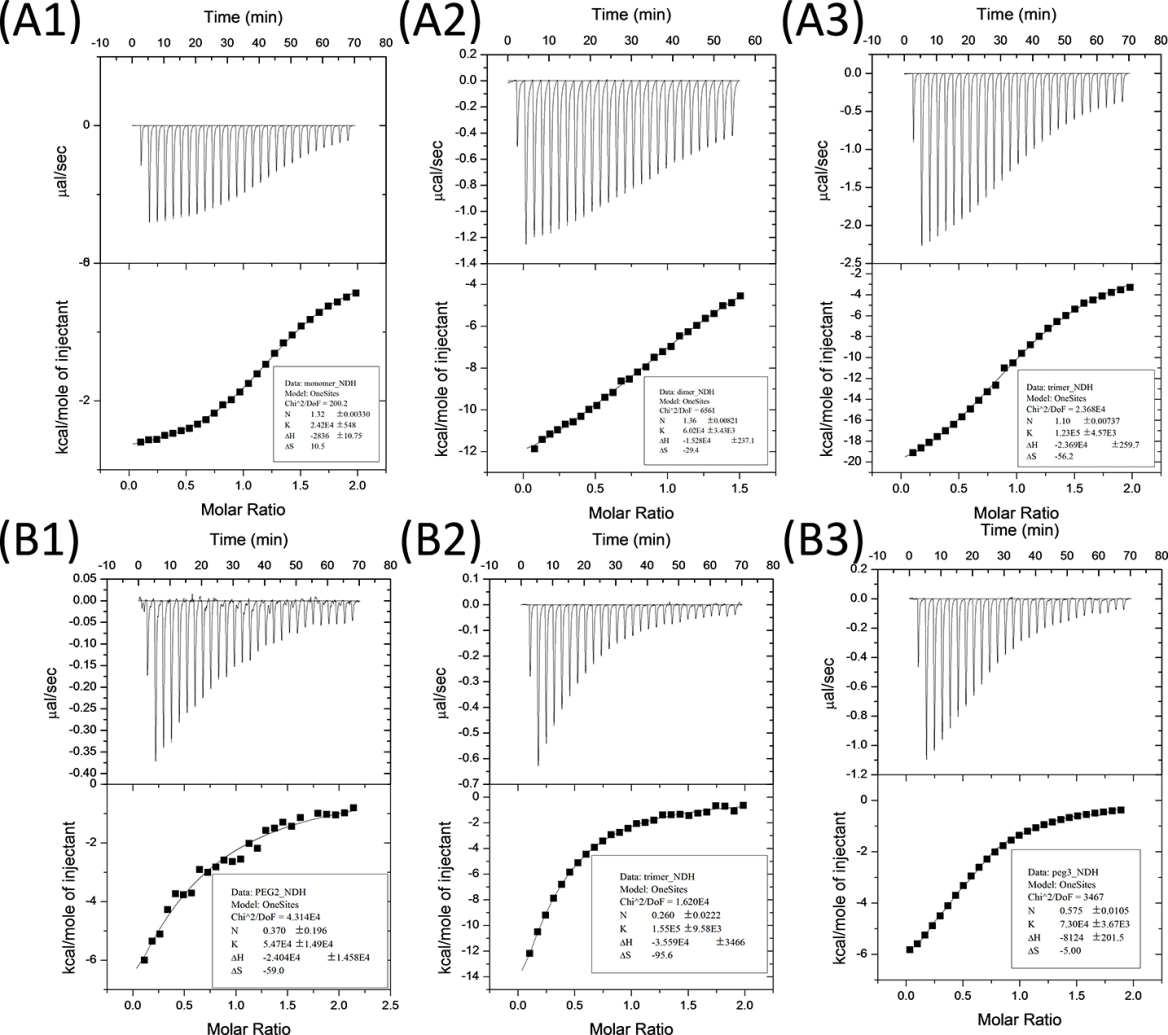
Isothermal titration calorimetry results and the curving fitting between peptide ligands and Shank1 PDZ protein. (A) The titration of ligands **p1**, **p2** and **p3** to Shank1 PDZ protein (A1, A2 and A3 are for **p1**, **p2** and **p3** respectively). (A1) [**p1**] = 4 mM, [Shank1 PDZ] = 400 μM; (A2) [**p2**] = 326 μM, [Shank1 PDZ] = 43 μM; (A3) [**p3**] = 354 μM, [Shank1 PDZ] = 35 μM. For the binding pair **p1** ligand and Shank1 PDZ, the affinity was very low so that protein concentration was adjusted higher to get accuracy affinity data. (B)The titration of diluted **p2**, **p3** and dimeric peptide with changed linker length to Shank1 PDZ protein. (B1) [**p2**] = 194 μM, [Shank1 PDZ] = 0.018 μM; (B2) [**p3**] = 160 μM, [Shank1 PDZ] = 16 μM; (B3) [dimeric peptide BM(PEG)_3_] = 600 μM, [Shank1 PDZ] = 53 μM.

**Fig 4:**
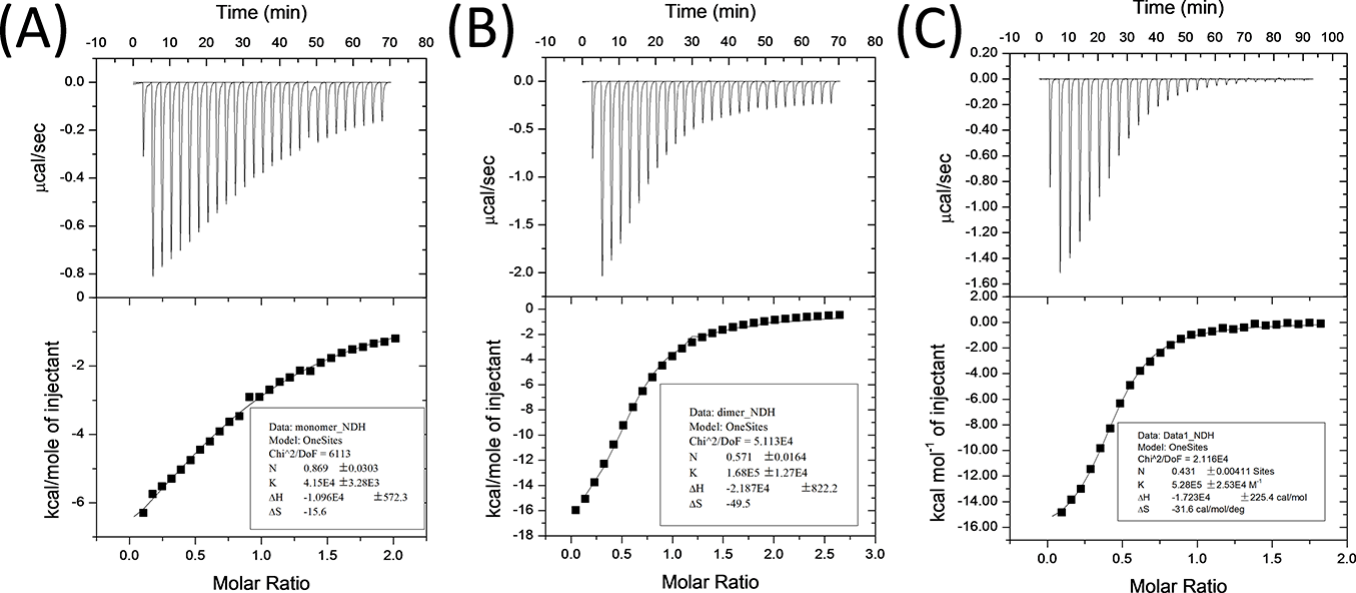
Isothermal titration calorimetry of ligands p1, p2 and p3 to Shank3 PDZ protein (A, B and C are for p1, p2 and p3 respectively). (A) [**p1**] = 406 μM, [Shank3 PDZ] = 40μM; (B) [**p2**] = 400 μM, [Shank3 PDZ] = 30 μM; (C) [**p3**] = 315 μM, [Shank3 PDZ] = 34 μM.

We then measured the thermodynamics of binding interaction between the ligand series and Shank1 PDZ protein by isothermal titration calorimetry. The PDZ domain of Shank1, one of the three family members of Shank proteins, Shank1 PDZ, was cloned, expressed, and purified to homogeneity for the binding studies (S1B and S1C Fig). The dissociation constants *K*_*d*_ derived by fitting the titration curves were found to be 41 ± 1 μM for monomeric peptide, 16.6 ± 0.9 μM for dimeric peptide, and 8.1 ± 0.3 μM for trimeric peptide (Fig 3A), indicating that dendrimeric peptides with higher density of PDZ-binding site also have higher binding affinity. Most of the *C* values (*C* = ([protein]/*K*_*d*_) × N; N is the fitted binding ratio value) of the curves are greater than 5, indicating that the *K*_*d*_ values measured here are highly reliable. In addition, to normalize the concentrations in ITC studies, we diluted the peptide dimer and trimer to see whether lower concentration will have influence on affinity enhancement. While too low concentration will lead to too low *C* value, and the *K*_*d*_ values will not be reliable. Therefore, we lowered the concentration of dimer to 194 μM and trimer to 160 μM, *K*_*d*_ were found to be 18 ± 5 μM for diluted dimer, and 6.5 ± 0.4 μM for diluted trimer (Fig 3B1 and 3B2), which were comparable with the previous data. Also by changing the linker length from 14.7 Å (PEG_2_) to 17.8 Å (PEG_3_), we found that *K*_*d*_ *=* 13.7 ± 0.7 μM (Fig 3B3), showing that linker length had slight while not significant influence on binding affinity. The same trend was also observed in a different PDZ protein, Shank3 PDZ, with the *K*_*d*_ values of the monomer, dimer, and trimer ligands with Shank3 PDZ measured to be 24 ± 2 μM, 5.9 ± 0.4 μM, and 1.9 ± 0.1 μM respectively (Fig 4). As Shank1 PDZ and Shank3 PDZ share pair-wise amino acid sequence identity close to 80% [18], this then confirms our hypothesis that clustered ligands bind with higher affinity than monovalent ligand. Therefore, clustering of protein-binding sites enhances the binding affinity, which explains the triple coiled-coil structure of βPIX attains higher binding affinity with Shank PDZ proteins to be more effectively anchored to synaptic site.

### 1:1 binding stoichiometry exhibited by all the peptide ligands with the receptor protein

As one peptide dimer or trimer can possibly bind to two or three PDZ proteins, we next performed native mass spectrometry analysis on the complexes to investigate the source of the affinity enhancement [19–22]. Analytical methods that accurately measure the binding stoichiometry without disturbing the complex are rare. Because of the small sizes of the peptide ligands, peptide−protein complex peaks cannot be differentiated from those of the proteins alone in size exclusion chromatography. Here we utilized mass spectrometry (MS) to study the protein-ligand interaction in gas phase, and demonstrated the gas-phase measurement can reveal the solution-phase binding characteristics. Electrospray ionization (ESI) transfers liquid-phase non-covalent complexes into the gas phase without disrupting the non-covalent interaction, allowing intact weakly bound complexes to be detected. Native MS analysis using ESI as a gentle ionization method allows determination of the assembly states, binding stoichiometry, and relative binding affinities [19–22].

**Fig 5:**
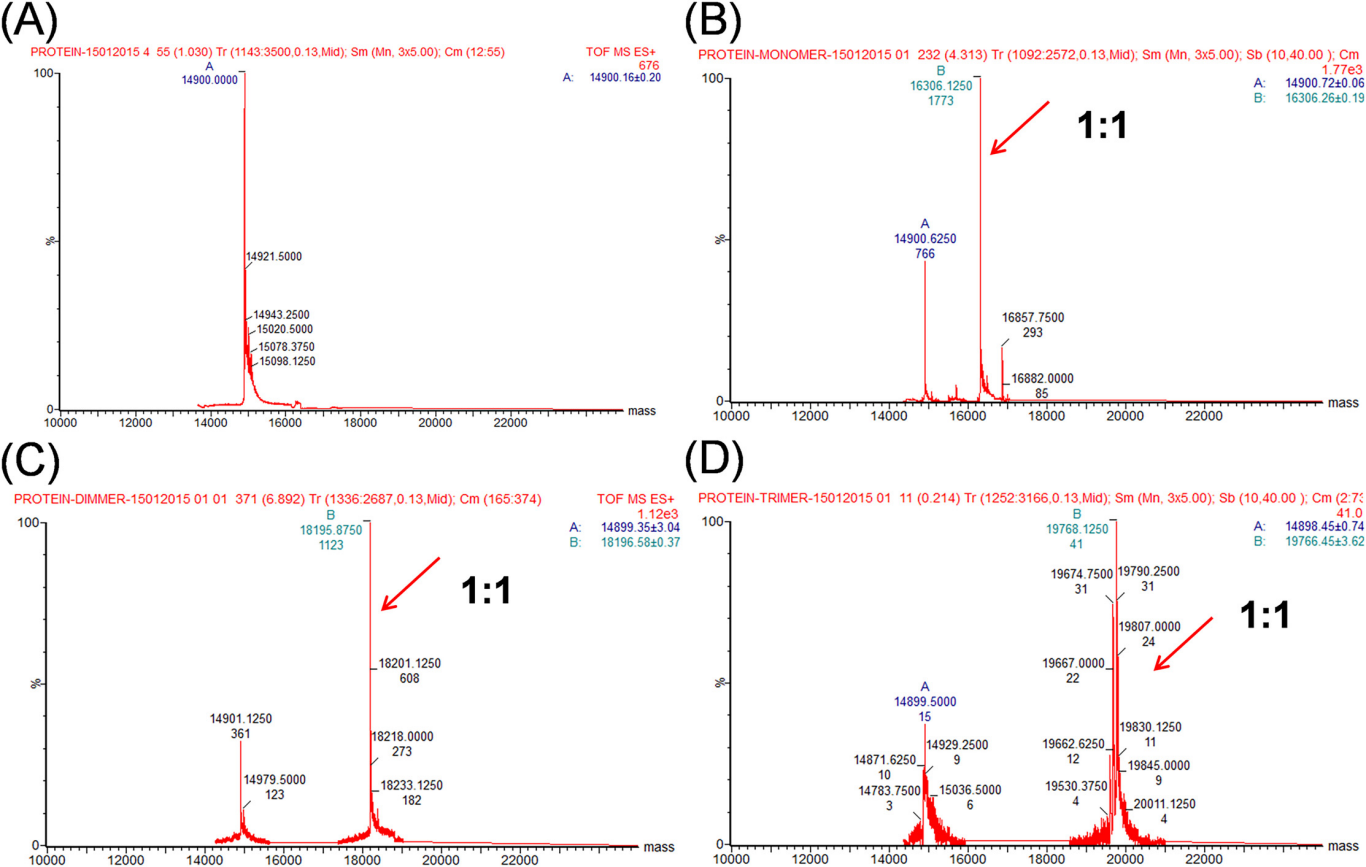
Native mass spectrometric analysis of Shank1 PDZ protein with peptide dendrimers in 1:1 mixture. (A) Deconvoluted native mass spectrum of Shank1 PDZ protein without ligands, 130 μM in 150 mM ammonium acetate. (B-D) Deconvoluted mass spectra of Shank1 PDZ protein and peptide ligands **p1**, **p2** and **p3** mixed at 1:1 ratio (B, C and D are for **p1**, **p2** and **p3** respectively). In all three spectra, protein−peptide complexes with 1:1 stoichiometry were observed (indicated by the arrows). [Shank1 PDZ] = [peptide] = 65 μM.

Because the well-folded globular proteins have relatively low surface charges, they often show narrow charge distribution at a low charge state in native mass spectra [22]. The same has been observed on Shank1 PDZ, indicating that the protein is well folded under the experimental condition (S3 Fig). We then examined the native mass spectra of protein/peptide mixtures, first at 1:1 ratio. For monomeric peptide **p1**, a new peak with molecular weight of 16306 emerged in the mixture, indicating the formation of protein−peptide complex with 1:1 stoichiometry. Similarly, peptide dimer **p2** formed a complex of 18195 in MW, and peptide trimer **p3** formed a complex with MW of 19768, all having the stoichiometry of 1:1 (Fig 5). On the other hand, as the peptide ligands are much smaller than the protein, we assume that ligand binding does not affect the protein ionization. The peak intensity in mass spectrum therefore corresponds to the concentration of the protein in the solution to the first order of approximation. We then estimated the dissociation constants to be roughly 13 μM for **p1**, 5.3 μM for **p2** and 4.5 μM for **p3**. Although being a rough estimation, the numbers are consistent with the trend in solution.

**Fig 6:**
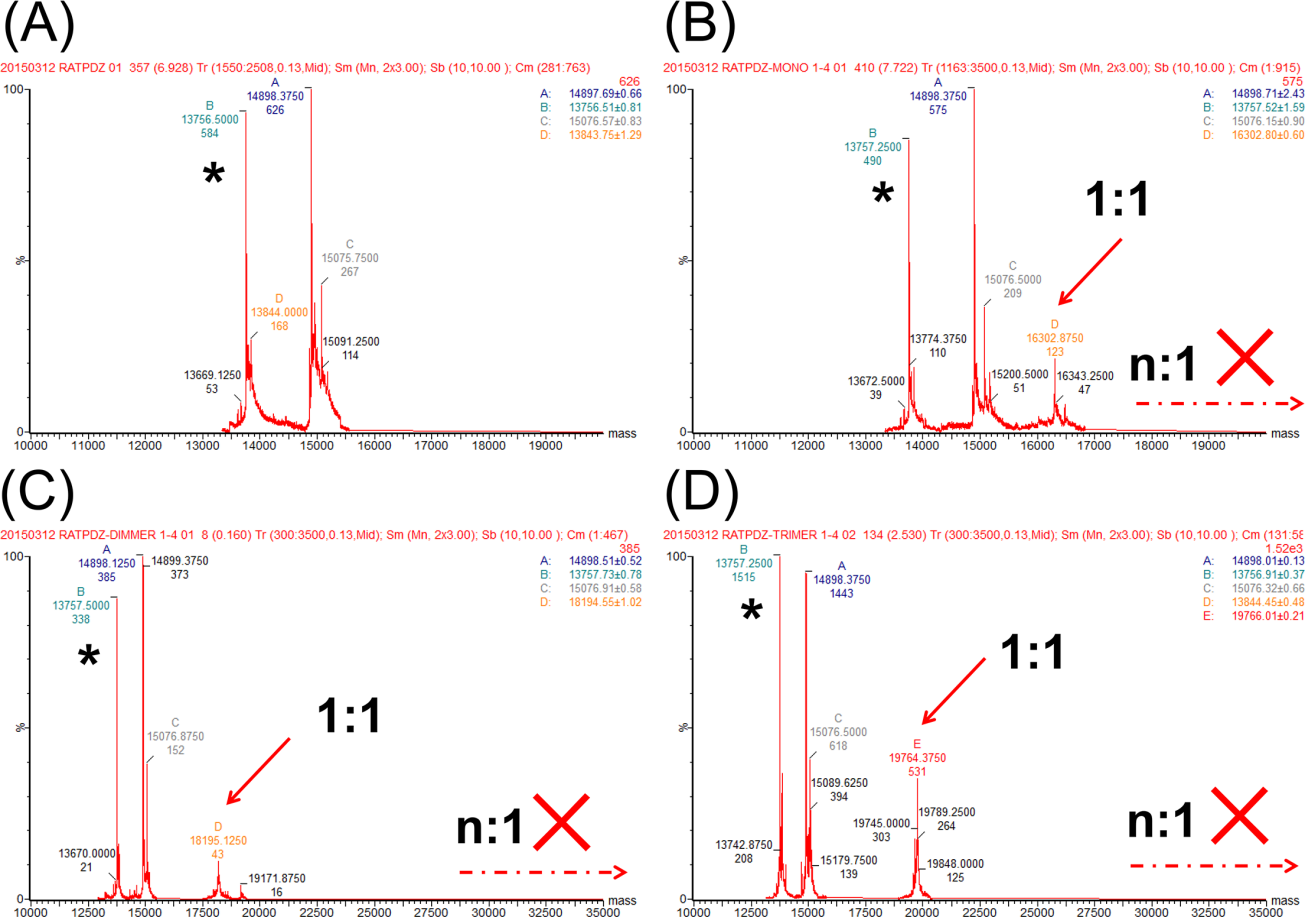
Native mass spectrometric analysis of Shank1 PDZ protein with peptide dendrimer in 4:1 mixture. (A) Deconvoluted native mass spectrum of Shank1 PDZ protein without ligands, 91 μM in 150 mM ammonium acetate. (B-D) Deconvoluted mass spectra of Shank1 PDZ protein and peptide ligands **p1**, **p2** and **p3** mixed at 4:1 ratio (B, C and D are for **p1**, **p2** and **p3** respectively). Similarly, only protein−peptide complexes with 1:1 stoichiometry were observed (indicated by the solid line arrows). [Shank1 PDZ] = 45 μM; [peptide] = 11 μM. The peak at 13757 (indicated by * in the spectra) is from an impurity protein that co-purified with Shank1 PDZ protein in this batch. The protein peaks at around 14900 is from the Shank PDZ protein with the first methionine removed and the peak at around 15076 is from the Shank PDZ protein with the first methionine; both peaks are present in the spectrum of protein itself, so they are not relevant to the protein−peptide complex.

We then examined the protein complexes in the mixture having protein/peptide ratio of 4:1 (protein in excess in order to promote multiple proteins to bind to one peptide dendrimer) by native mass spectrometry. Still, only protein−peptide complexes with 1:1 stoichiometry were observed on mass spectra; but complexes peaks with higher stoichiometry such as 2:1 or 3:1 were not observed for peptide dimer and trimer in the expected mass range (high MW range in Fig 6), although protein was in excess. Therefore, within the detection limit of native MS, this evidence provides explicit support that the dendrimeric peptides bind with PDZ protein only with 1:1 stoichiometry.

### Decreased dissociation rate constant resulted in enhanced binding affinity

**Fig 7:**
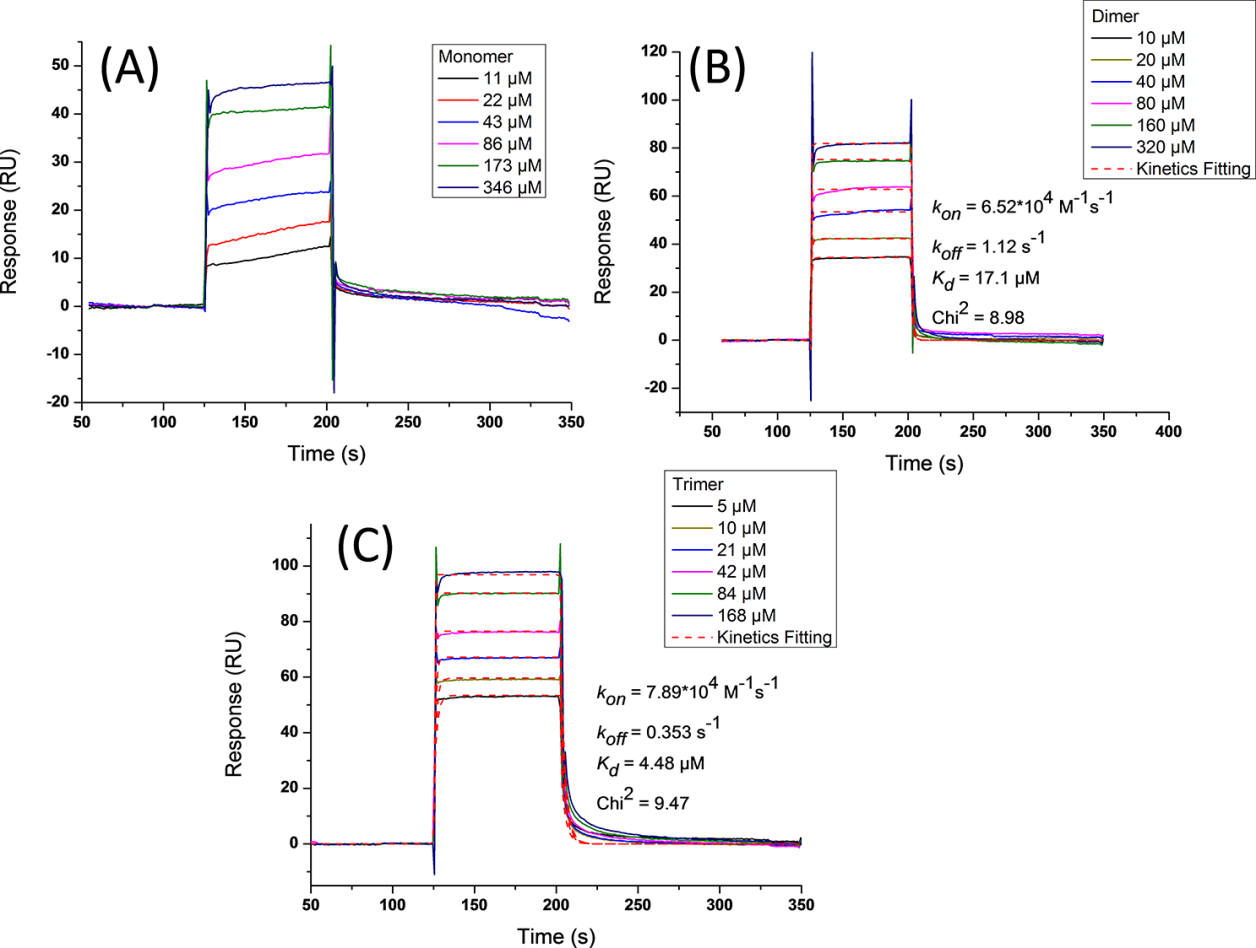
Surface plasmon resonance demonstrated ligands p1, p2 and p3 binding to Shank1 PDZ protein immobilized on the sensor chip. (A, B and C are for **p1**, **p2** and **p3** respectively). (A) [**p1**] = 11–346 μM; (B) [**p2**] = 10–320 μM; (C) [**p3**] = 5–168 μM. Dash lines in (B) & (C) were kinetics fitting used 1:1 Langmuir binding model for dimer **p2** and trimer **p3**.

**Fig 8:**
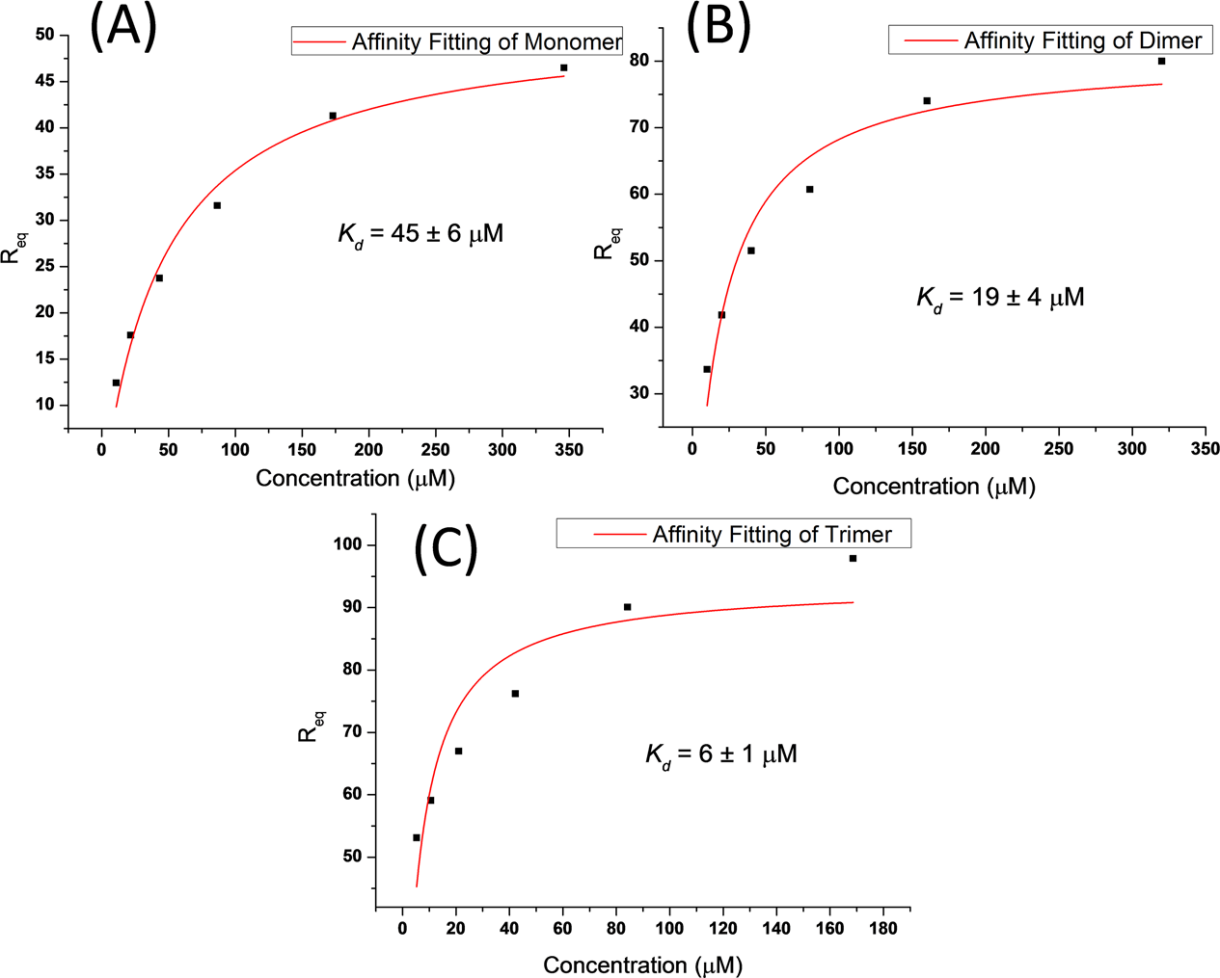
*R*_*eq*_ results from surface plasmon resonance and the curving fitting of ligand p1, p2 and p3 to Shank1 PDZ protein (Figure A, B, and C for ligand p1, p2 and p3 respectively). Fitting followed the steady state affinity model using this equation: $R_{eq}\;=\;\frac{K_{a}CR_{max}}{1+\;K_{a}Cn}$ here *R*_*eq*_ referred to response on the sensorgrams in the steady state region of the curve, *C* was concentration of analyte, *R*_*max*_ was the theoretical binding capacity, *n* = 1 in 1:1 binding model, *K*_*a*_ was association constant, and *K*_*d*_ could be obtained through 1/ *K*_*a*_.

To evaluate the influence factor behind the binding enhancement, we assessed the kinetic parameters using surface plasmon resonance (SPR). SPR sensograms showed concentration-dependent binding of peptide ligands to Shank1 PDZ immobilized on the sensor chip. All the three ligands displayed favourable binding kinetics with fast on-rate and fast off-rate model (Fig 7). Therefore, we first evaluated affinity using steady state affinity model. The fitting results showed dissociation constants of 45 ± 6 μM for ligand **p1**, 19 ± 4 μM for ligand **p2**, 6 ± 1 μM for ligand **p3**(Fig 8), which were essentially in agreement with the affinity data attained in ITC. Next, we successful got kinetics data for ligands **p2** (Fig 7B) and **p3** (Fig 7C) to Shank1 PDZ protein using 1:1 Langmuir binding model, although fitting for fast on-rate and fast off-rate model was very difficult. For ligand **p1**, kinetics data couldn’t be attained because kinetics fitting wasn’t always suitable for fast on-rate and fast off-rate model. The association rate constant *k*_*on*_ was 6.52*10^4^ M^-1^s^-1^ for ligand **p2** and 7.89*10^4^ M^-1^s^-1^ for ligand **p3**, and the dissociation rate constant *k*_*off*_ was 1.12 s^-1^ for ligand **p2** and 0.353 s^-1^ for ligand **p3**. At the meanwhile, the affinities gotten in kinetics fitting (17.1 μM for ligand **p2**, 4.48 μM for ligand **p3**) were comparable to affinity model. The rate constant clearly showed small difference in *k*_*on*_ (about 1.2 fold) while large difference in *k*_*off*_ (about 3.1 fold), indicating that decreased dissociation rate constant dominantly resulted in enhanced binding affinity. This binding profile suggested the proximity effect played a key role in the binding enhancement, which also proved by the native MS experiment.

Taken together, ITC, native MS and SPR analyses revealed that peptide trimer binds Shank PDZ with higher affinity than peptide dimer, and peptide dimer binds tighter than peptide monomer. Such affinity enhancement was not achieved through binding two or three proteins to one ligand, because protein−peptide complexes with 1:1 stoichiometry were the only species we observed in native MS. In addition, difference in dissociation rate constant *k*_*off*_ was larger than difference in association rate constant *k*_*on*_. Both characters demonstrated that this affinity enhancement observed here can be explained that proximity of multiple protein-binding sites in a limited space increases the local concentration of binding ligands, which then increases the chance that the dissociated protein to re-bind with one of the protein-binding sites [23, 24]. This effect observed in peptide dendrimer recapitulates that of the binding between Shank PDZ and βPIX trimer: βPIX likely trimerizes through a parallel coiled coil to achieve high local concentration of PDZ-binding peptide, and thereby more stable synaptic anchoring can be achieved.

## Conclusions

Besides shedding light on βPIX−Shank PDZ interaction, affinity enhancement through ligand clustering might also provide hint to the design of high affinity inhibitors for PDZ proteins [25]. Inhibitors with multiple protein-binding sites will bind with the protein target with higher affinity and greater specificity than the endogenous binding partner, which will find value in effective inhibition of protein−protein interaction inside cells. Such explorations are underway.

## Supporting Information

S1 Fig Expression and purification of Shank proteins. (A) Recombinant plasmid of pET28 Shank1 PDZ. (B) SDS-PAGE of purified Shank1 PDZ. (C) SDS-PAGE of purified Shank3 PDZ. (D) Gel filtration chromatography profile of purified Shank1 PDZ. (E) Gel filtration chromatography profile of purified Shank3 PDZ.
(TIF)

S2 Fig **Identity of dendritic ligands: monomeric ligand p1 (A), dimeric ligand p2 (B), trimeric ligand p3 (C).** (A1) HPLC trace of **p1**; (A2) Mass spectra of **p1**: theoretical [M+2H]^2+^: 703.2970, experimental [M+2H]^2+^: 703.2971; (B1) HPLC trace of **p2;** (B2) Mass spectra of **p2**: theoretical [M+2H]^2+^: 1648.1368, experimental [M+2H]^2+^: 1648.1308; (C1) HPLC trace of **p3;** (C2) Mass spectra of **p3**: [M+3H]^3+^: 1622.9609, experimental [M+3H]^3+^: 1622.9551.
(TIF)

S3 Fig Native mass spectrum of Shank1 PDZ protein without ligands (130 μM in 150 mM ammonium acetate). (TIF)
