# Supplementary figures and images for: Affinity Enhancement by Ligand Clustering Effect Inspired by Peptide Dendrimers−Shank PDZ Proteins Interactions
Source: PLoS One. 2016 Feb 26;11(2):e0149580. doi: 10.1371/journal.pone.0149580 (PMC4769301; doi:10.1371/journal.pone.0149580)

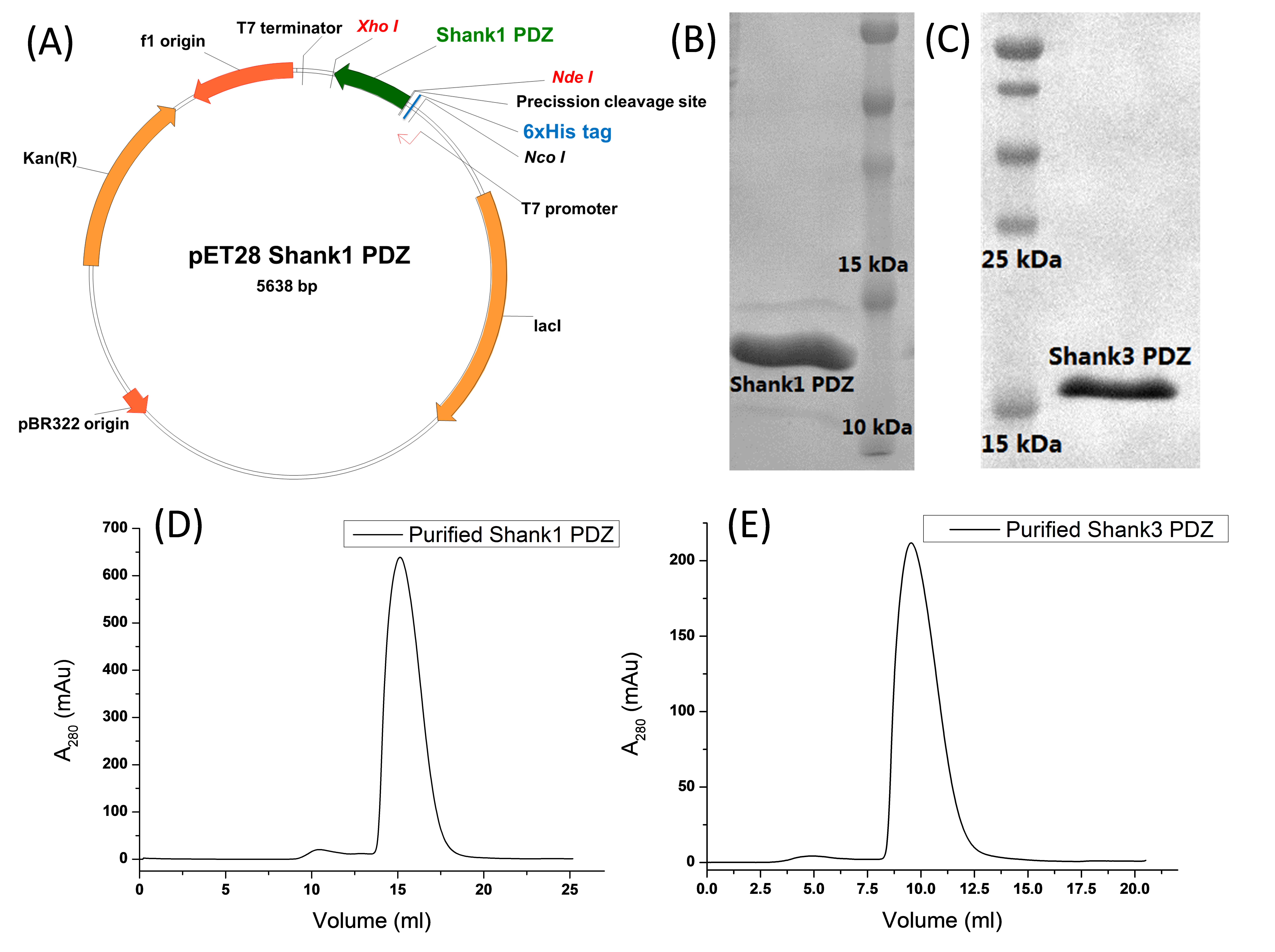

Supplement: S1 Fig — (A) Recombinant plasmid of pET28 Shank1 PDZ. (B) SDS-PAGE of purified Shank1 PDZ. (C) SDS-PAGE of purified Shank3 PDZ. (D) Gel filtration chromatography profile of purified Shank1 PDZ. (E) Gel filtration chromatography profile of purified Shank3 PDZ. (TIF) [file pone.0149580.s001.tif]

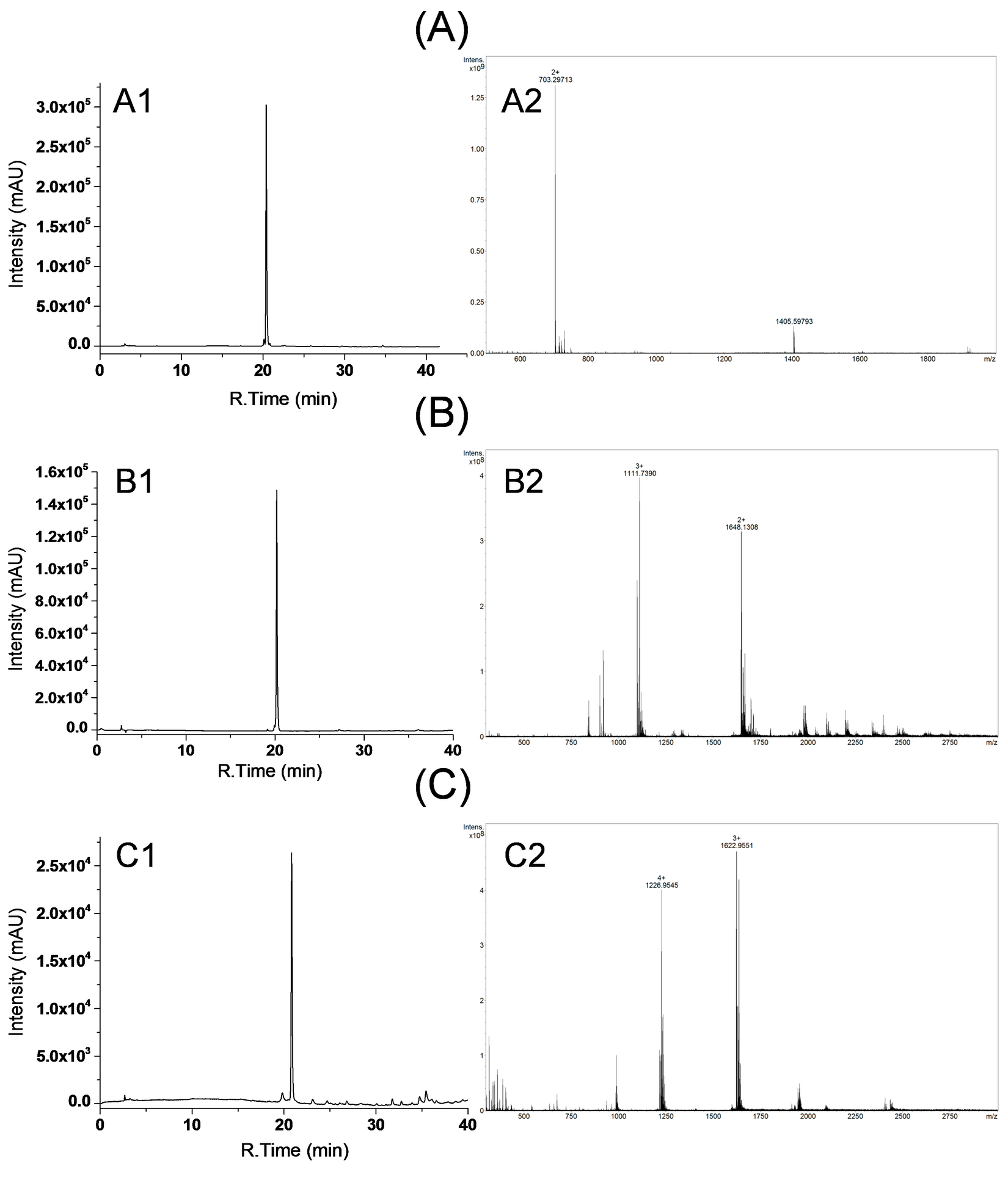

Supplement: S2 Fig — Identity of dendritic ligands: monomeric ligand p1 (A), dimeric ligand p2 (B), trimeric ligand p3 (C). (A1) HPLC trace of p1; (A2) Mass spectra of p1: theoretical [M+2H]2+: 703.2970, experimental [M+2H]2+: 703.2971; (B1) HPLC trace of p2; (B2) Mass spectra of p2: theoretical [M+2H]2+: 1648.1368, experimental [M+2H]2+: 1648.1308; (C1) HPLC trace of p3; (C2) Mass spectra of p3: [M+3H]3+: 1622.9609, experimental [M+3H]3+: 1622.9551. (TIF) [file pone.0149580.s002.tif]

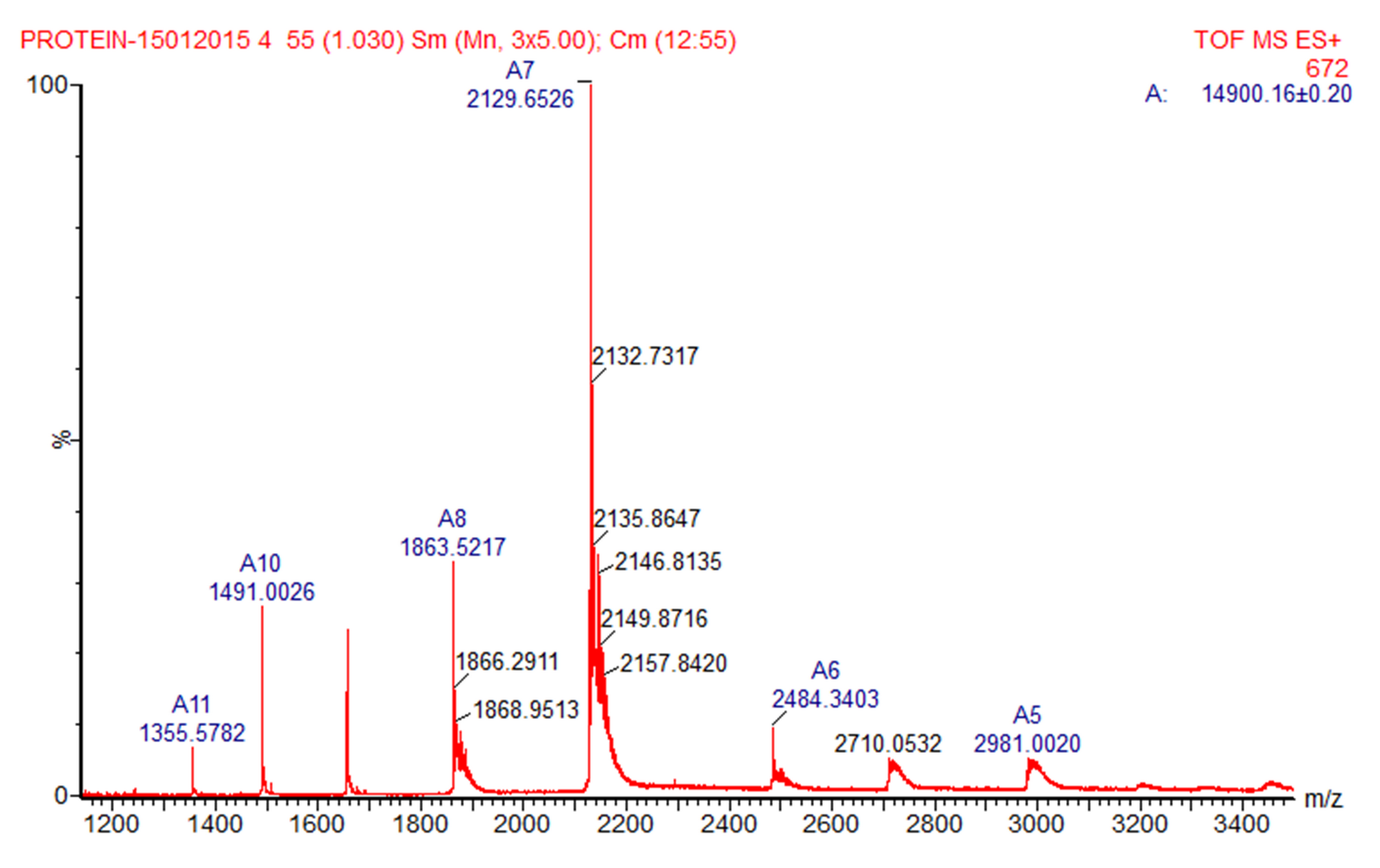

Supplement: S3 Fig — (TIF) [file pone.0149580.s003.tif]
